# Supplementary material for: Deep neural networks detect regional wall motion abnormalities and preclinical cardiovascular disease from 12-lead ECGs
Source: medRxiv. 2024 Jun 1:2024.05.31.24308304. Preprint. [Version 1] doi: 10.1101/2024.05.31.24308304 (PMC11160848; doi:10.1101/2024.05.31.24308304)
Supplement: 1 [file NIHPP2024.05.31.24308304V1-supplement-1.pdf]

medRxiv preprint doi: https://doi.org/10.1101/2024.05.31.24308304; this version posted June 1, 2024. The copyright holder for this preprint (which was not certified by peer review) is the author/funder, who has granted medRxiv a license to display the preprint in perpetuity.
It is made available under a CC-BY 4.0 International license .

**Supplementary Figure 1**. Left ventricular wall segme labeling convention for each of the seven ASE regions[14].

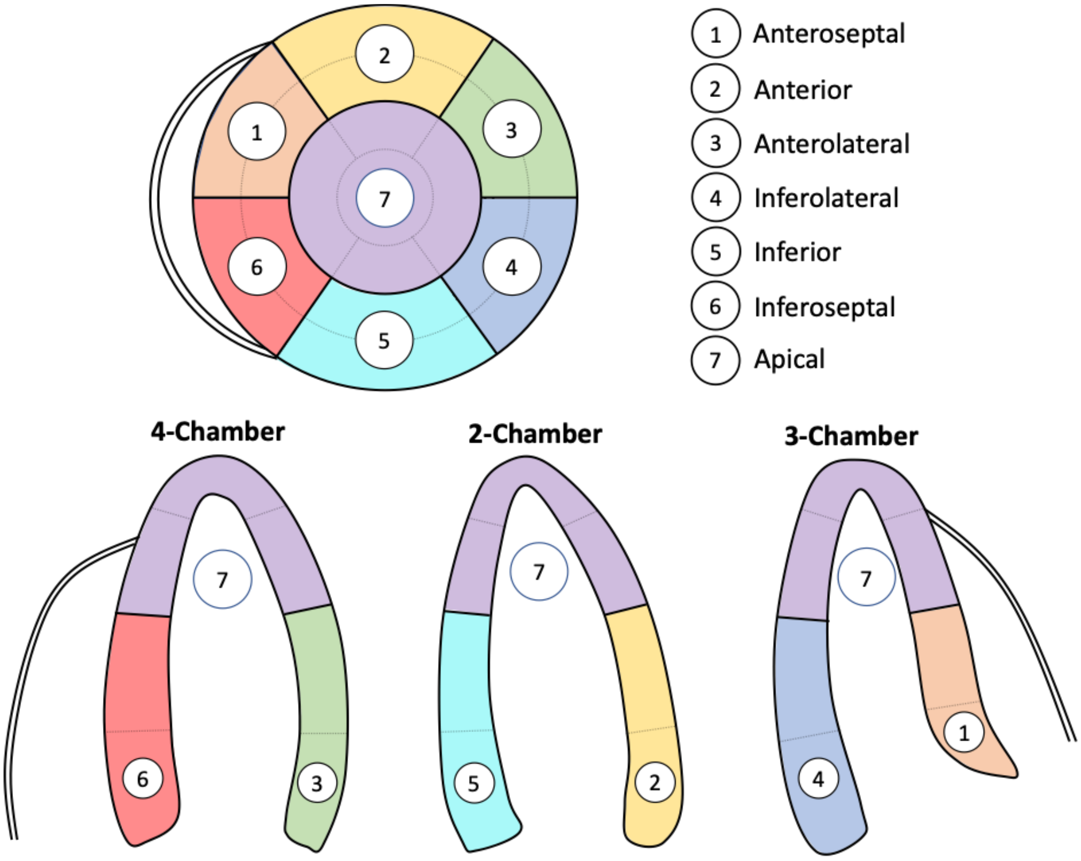


| Label | Convention |
|---|---|
| Anteroseptal | basal anteroseptal, mid anteroseptal |
| Anterior | basal anterior, mid anterior |
| Anterolateral | basal anterolateral, mid anterolateral, lateral |
| Inferolateral | basal inferolateral, mid inferolateral, posterior |
| Inferior | basal inferior, mid inferior |
| Inferoseptal | basal inferoseptal, mid inferoseptal, septal |
| Apical | apex, septal apex, lateral apex, inferior apex, anterior apex |

medRxiv preprint doi: https://doi.org/10.1101/2024.05.31.24308304; this version posted June 1, 2024. The copyright holder for this preprint
(which was not certified by peer review) is the author/funder, who has granted medRxiv a license to display the preprint in perpetuity.
It is made available under a CC-BY 4.0 International license .

**Supplementary Table 1.** ECG findings from the entire cohort. Values are provided as n (%). Chi-squared test was used.

| | RWMA | | | RV hypokinesis | | | LVEF ≤40% | | |
|---|---|---|---|---|---|---|---|---|---|
| | **Control** | **Case** | **P value** | **Control** | **Case** | **P value** | **Control** | **Case** | **P value** |
| **Primary rhythm** | | | | | | | | | |
| **Normal sinus rhythm** | 37418 (53.0) | 14810 (58.6) | <0.001 | 43476 (56.4) | 4007 (41.4) | <0.001 | 39684 (55.4) | 11853 (51.7) | <0.001 |
| **Atrial fibrillation** | 11699 (16.6) | 3374 (13.3) | <0.001 | 11107 (14.4) | 2527 (26.1) | <0.001 | 10650 (14.9) | 4176 (18.2) | <0.001 |
| **Sinus tachycardia** | 9828 (13.9) | 2539 (10.0) | <0.001 | 9850 (12.8) | 1205 (12.5) | 0.401 | 9363 (13.1) | 2813 (12.3) | 0.002 |
| **Sinus bradycardia** | 5665 (8.0) | 1906 (7.5) | 0.016 | 6568 (8.5) | 436 (4.5) | <0.001 | 6447 (9.0) | 1054 (4.6) | <0.001 |
| **Atrial flutter** | 1968 (2.8) | 489 (1.9) | <0.001 | 1726 (2.2) | 460 (4.8) | <0.001 | 1714 (2.4) | 701 (3.1) | <0.001 |
| **Atrial tachycardia** | 937 (1.3) | 251 (1.0) | <0.001 | 849 (1.1) | 193 (2.0) | <0.001 | 806 (1.1) | 361 (1.6) | <0.001 |
| **Ectopic atrial rhythm** | 801 (1.1) | 334 (1.3) | 0.02 | 900 (1.2) | 102 (1.1) | 0.357 | 847 (1.2) | 274 (1.2) | 0.906 |
| **Junctional rhythm** | 709 (1.0) | 284 (1.1) | 0.115 | 702 (0.9) | 181 (1.9) | <0.001 | 735 (1.0) | 235 (1.0) | 0.982 |
| **Supraventricular tachycardia** | 572 (0.8) | 160 (0.6) | 0.006 | 509 (0.7) | 127 (1.3) | <0.001 | 516 (0.7) | 208 (0.9) | 0.005 |
| **Ventricular rhythm** | 271 (0.4) | 134 (0.5) | 0.002 | 276 (0.4) | 79 (0.8) | <0.001 | 277 (0.4) | 115 (0.5) | 0.022 |
| **Wide QRS tachycardia** | 160 (0.2) | 120 (0.5) | <0.001 | 169 (0.2) | 70 (0.7) | <0.001 | 101 (0.1) | 173 (0.8) | <0.001 |
| **Ventricular tachycardia** | 126 (0.2) | 112 (0.4) | <0.001 | 151 (0.2) | 54 (0.6) | <0.001 | 79 (0.1) | 150 (0.7) | <0.001 |
| **Junctional ectopic rhythm** | 146 (0.2) | 63 (0.2) | 0.244 | 160 (0.2) | 31 (0.3) | 0.034 | 161 (0.2) | 45 (0.2) | 0.469 |
| **Secondary rhythm** | | | | | | | | | |
| **Premature ventricular complex(es)** | 5634 (8.0) | 2923 (11.6) | <0.001 | 6134 (8.0) | 1544 (16.0) | <0.001 | 4980 (7.0) | 3406 (14.9) | <0.001 |
| **Premature supraventricular complex(es)** | 4356 (6.2) | 1449 (5.7) | 0.013 | 4615 (6.0) | 538 (5.6) | 0.105 | 4361 (6.1) | 1365 (6.0) | 0.466 |
| **Axis deviation** | | | | | | | | | |
| **Left axis deviation** | 6551 (9.3) | 2877 (11.4) | <0.001 | 7291 (9.5) | 1142 (11.8) | <0.001 | 6376 (8.9) | 2933 (12.8) | <0.001 |
| **Right axis deviation** | 1442 (2.0) | 386 (1.5) | <0.001 | 1135 (1.5) | 512 (5.3) | <0.001 | 1369 (1.9) | 431 (1.9) | 0.783 |
| **Atrioventricular conduction delay** | | | | | | | | | |
| **First-degree AV block** | 5568 (7.9) | 2630 (10.4) | <0.001 | 6373 (8.3) | 997 (10.3) | <0.001 | 5653 (7.9) | 2437 (10.6) | <0.001 |
| **High-grade AV block** | 252 (0.4) | 84 (0.3) | 0.615 | 226 (0.3) | 65 (0.7) | <0.001 | 222 (0.3) | 111 (0.5) | <0.001 |
| **Complete heart block** | 135 (0.2) | 48 (0.2) | 0.964 | 141 (0.2) | 24 (0.2) | 0.205 | 140 (0.2) | 42 (0.2) | 0.778 |
| **Ventricular hypertrophy** | | | | | | | | | |
| **Left ventricular hypertrophy** | 6192 (8.8) | 2496 (9.9) | <0.001 | 7206 (9.3) | 818 (8.5) | 0.005 | 6099 (8.5) | 2473 (10.8) | <0.001 |

medRxiv preprint doi: https://doi.org/10.1101/2024.05.31.24308304; this version posted June 1, 2024. The copyright holder for this preprint (which was not certified by peer review) is the author/funder, who has granted medRxiv a license to display the preprint in perpetuity.
It is made available under a CC-BY 4.0 International license .

| | | | | | | | | | |
|---|---|---|---|---|---|---|---|---|---|
| **Right ventricular hypertrophy** | 321 (0.5) | 54 (0.2) | <0.001 | 207 (0.3) | 135 (1.4) | <0.001 | 307 (0.4) | 64 (0.3) | 0.002 |
| **Intraventricular conduction delay** | | | | | | | | | |
| **Right bundle branch block** | 5676 (8.0) | 2496 (9.9) | <0.001 | 5905 (7.7) | 1344 (13.9) | <0.001 | 5839 (8.2) | 2191 (9.6) | <0.001 |
| **Left anterior fascicular block** | 4425 (6.3) | 1985 (7.9) | <0.001 | 4898 (6.3) | 815 (8.4) | <0.001 | 4445 (6.2) | 1859 (8.1) | <0.001 |
| **Idioventricular rhythm** | 3569 (5.1) | 2116 (8.4) | <0.001 | 4116 (5.3) | 935 (9.7) | <0.001 | 3095 (4.3) | 2469 (10.8) | <0.001 |
| **Left bundle branch block** | 3316 (4.7) | 2022 (8.0) | <0.001 | 4056 (5.3) | 715 (7.4) | <0.001 | 2332 (3.3) | 2890 (12.6) | <0.001 |
| **Left posterior fascicular block** | 166 (0.2) | 68 (0.3) | 0.386 | 155 (0.2) | 51 (0.5) | <0.001 | 168 (0.2) | 66 (0.3) | 0.181 |
| **Waveform abnormalities** | | | | | | | | | |
| **Non-specific ST-T abnormality** | 17784 (25.2) | 5192 (20.5) | <0.001 | 18602 (24.1) | 2260 (23.4) | 0.109 | 18094 (25.3) | 4607 (20.1) | <0.001 |
| **Artifact** | 7733 (10.9) | 2348 (9.3) | <0.001 | 8175 (10.6) | 951 (9.8) | 0.022 | 7843 (11.0) | 2099 (9.2) | <0.001 |
| **ST-T abnormality** | 7112 (10.1) | 2957 (11.7) | <0.001 | 8252 (10.7) | 881 (9.1) | <0.001 | 7641 (10.7) | 2304 (10.0) | 0.008 |
| **P wave abnormality** | 5934 (8.4) | 2442 (9.7) | <0.001 | 6620 (8.6) | 967 (10.0) | <0.001 | 5798 (8.1) | 2431 (10.6) | <0.001 |
| **QTc prolongation** | 4314 (6.1) | 1838 (7.3) | <0.001 | 4929 (6.4) | 663 (6.9) | 0.082 | 4491 (6.3) | 1587 (6.9) | <0.001 |
| **ST elevation** | 1543 (2.2) | 2062 (8.2) | <0.001 | 2833 (3.7) | 329 (3.4) | 0.191 | 2172 (3.0) | 1363 (5.9) | <0.001 |
| **Myocardial ischemia** | | | | | | | | | |
| **MI of indeterminate age** | 6205 (8.8) | 4897 (19.4) | <0.001 | 8442 (10.9) | 1396 (14.4) | <0.001 | 7009 (9.8) | 3867 (16.9) | <0.001 |
| **Ischemia pattern** | 4679 (6.6) | 3018 (11.9) | <0.001 | 6019 (7.8) | 927 (9.6) | <0.001 | 5132 (7.2) | 2469 (10.8) | <0.001 |
| **Old myocardial infarction** | 4488 (6.4) | 2819 (11.2) | <0.001 | 5588 (7.2) | 949 (9.8) | <0.001 | 4742 (6.6) | 2416 (10.5) | <0.001 |
| **Subacute myocardial infarction** | 3535 (5.0) | 2693 (10.7) | <0.001 | 4797 (6.2) | 775 (8.0) | <0.001 | 3906 (5.5) | 2194 (9.6) | <0.001 |
| **Pacemaker** | | | | | | | | | |
| **Ventricular pacemaker** | 2530 (3.6) | 1521 (6.0) | <0.001 | 2750 (3.6) | 836 (8.6) | <0.001 | 1943 (2.7) | 1985 (8.7) | <0.001 |
| **Atrial pacemaker** | 736 (1.0) | 390 (1.5) | <0.001 | 857 (1.1) | 138 (1.4) | 0.007 | 709 (1.0) | 402 (1.8) | <0.001 |

medRxiv preprint doi: https://doi.org/10.1101/2024.05.31.24308304; this version posted June 1, 2024. The copyright holder for this preprint (which was not certified by peer review) is the author/funder, who has granted medRxiv a license to display the preprint in perpetuity.
It is made available under a CC-BY 4.0 International license .

**Supplementary Table 2.** ICD-9 and ICD-10 codes used for MIMIC-IV.

| Comorbidity | ICD-9 | ICD-10 |
|---|---|---|
| Congestive heart failure | 398.91, 402.01, 402.11, 402.91, 404.01, 404.03, 404.11, 404.13, 404.91, 404.93, 425.4-425.9, 428 | I11.0, I13.0, I13.2, I25.5, I42.0, I42.5-I42.9, I50 |
| Myocardial infarction | 410, 412 | I21, I22, I25.2 |
| Hypertension | 401, 402, 403, 404, 405, 642 | I10, I11, I12, I13, I14, I15 |
| Coronary artery disease | 410, 411, 412, 414 | I21, I22, I23, I24, 125 |
| Diabetes | 250, 357.2, 362.0, 366.41 | E10.0–E14.91 |

medRxiv preprint doi: https://doi.org/10.1101/2024.05.31.24308304; this version posted June 1, 2024. The copyright holder for this preprint
(which was not certified by peer review) is the author/funder, who has granted medRxiv a license to display the preprint in perpetuity.
It is made available under a CC-BY 4.0 International license .

**Supplementary Table 3.** Number of wall motion findings by left ventricular region across the entire cohort.

| | Normal | Hypokinesis | Akinesis | Dyskinesis |
|---|---|---|---|---|
| **Anterior wall** | 23192 (93.6%) | 954 (3.8%) | 580 (2.3%) | 64 (0.3%) |
| **Anteroseptal wall** | 22944 (92.6%) | 1214 (4.9%) | 594 (2.4%) | 38 (0.2%) |
| **Inferoseptal wall** | 23114 (93.2%) | 1304 (5.3%) | 355 (1.4%) | 17 (0.1%) |
| **Anterolateral wall** | 23790 (96.0%) | 881 (3.6%) | 118 (0.5%) | 1 (0.0%) |
| **Inferolateral wall** | 22096 (89.1%) | 1622 (6.5%) | 1003 (4.0%) | 69 (0.3%) |
| **Inferior wall** | 21455 (86.5%) | 1886 (7.6%) | 1336 (5.4%) | 113 (0.5%) |
| **Apex** | 21931 (88.5%) | 1528 (6.2%) | 966 (3.9%) | 365 (1.5%) |

medRxiv preprint doi: https://doi.org/10.1101/2024.05.31.24308304; this version posted June 1, 2024. The copyright holder for this preprint (which was not certified by peer review) is the author/funder, who has granted medRxiv a license to display the preprint in perpetuity.
It is made available under a CC-BY 4.0 International license .

**Supplementary Table 4.** Performance of the reference model on the test set to identify the presence of RWMA.

| | **AUC** | **Sensitivity** | **Specificity** | **PPV** | **NPV** |
|---|---|---|---|---|---|
| **ECG reports** | 0.696 | 76.4 | 47.9 | 35.6 | 47.9 |
| **ECG reports + age + sex** | 0.703 | 78.4 | 48.4 | 36.4 | 48.4 |

medRxiv preprint doi: https://doi.org/10.1101/2024.05.31.24308304; this version posted June 1, 2024. The copyright holder for this preprint (which was not certified by peer review) is the author/funder, who has granted medRxiv a license to display the preprint in perpetuity.
It is made available under a CC-BY 4.0 International license .

**Supplementary Figure 2.** Sensitivity of the deep learning model on the test set to identify the presence of RWMA based on the number of regions with abnormal wall motion.

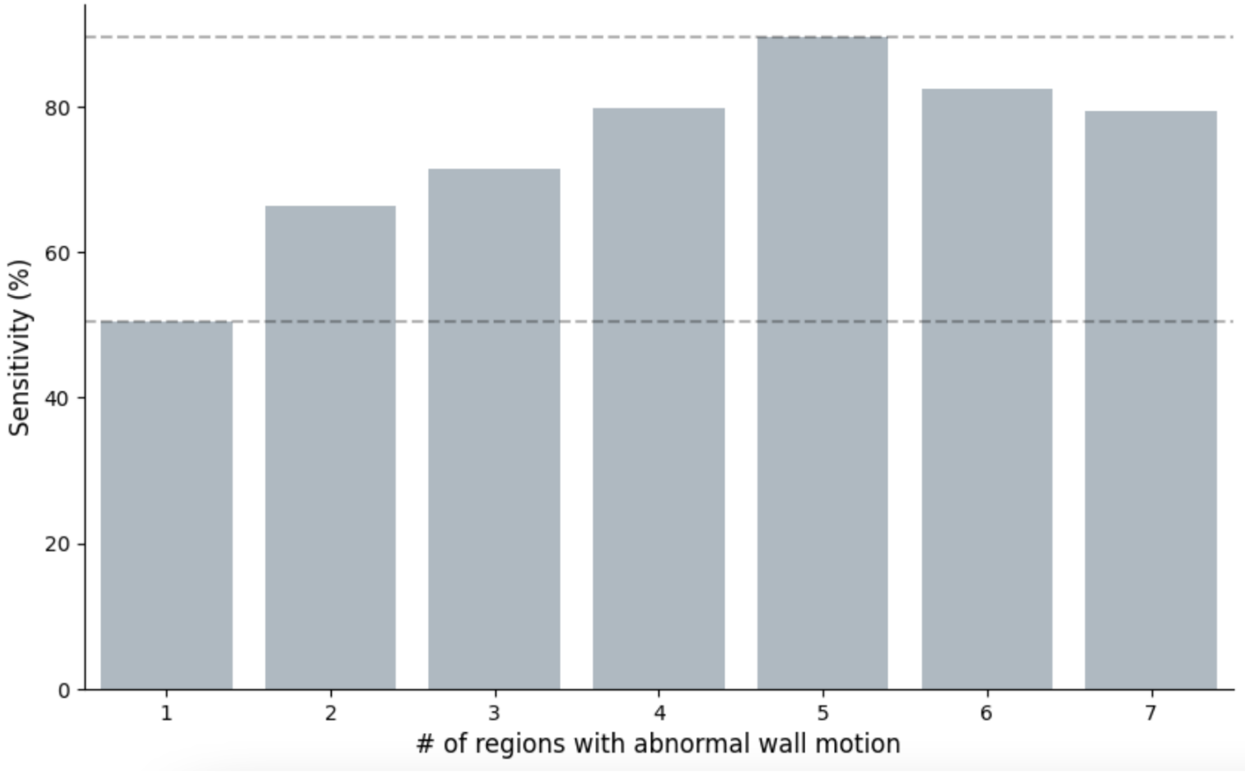

medRxiv preprint doi: https://doi.org/10.1101/2024.05.31.24308304; this version posted June 1, 2024. The copyright holder for this preprint (which was not certified by peer review) is the author/funder, who has granted medRxiv a license to display the preprint in perpetuity.
It is made available under a CC-BY 4.0 International license .

**Supplementary Figure 3.** Subgroup analysis by ECG findings extracted from cardiologist reports in MIMIC-IV-ECG using diagnostic odds ratio (OR) with 95% confidence intervals (CIs). The vertical dashed lines represent the OR of the deep learning model across all patients in the test set.

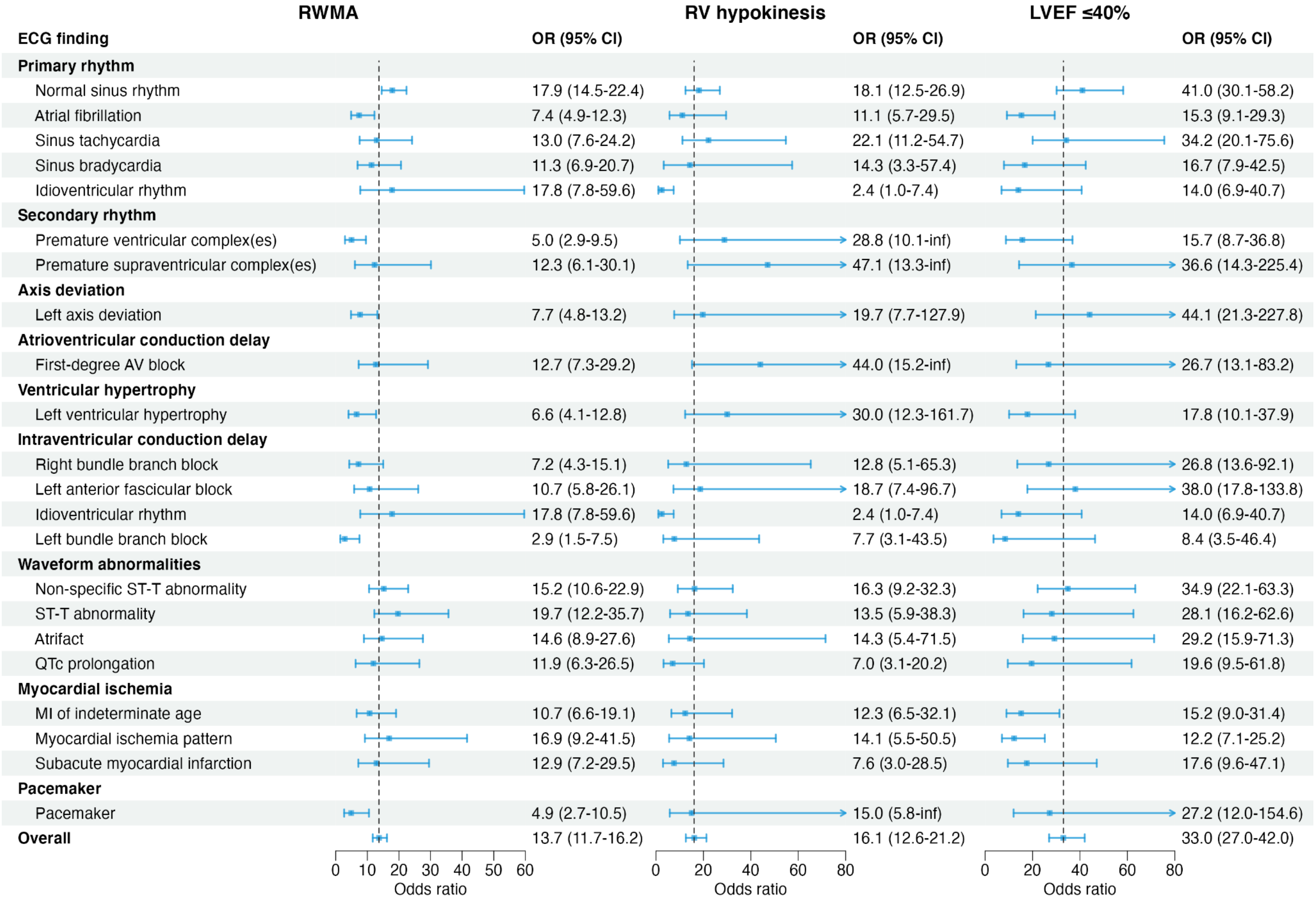

medRxiv preprint doi: https://doi.org/10.1101/2024.05.31.24308304; this version posted June 1, 2024. The copyright holder for this preprint (which was not certified by peer review) is the author/funder, who has granted medRxiv a license to display the preprint in perpetuity.
It is made available under a CC-BY 4.0 International license .

## *Appendix*

### *Training details*

The deep learning model was pre-trained using 235,568 ECGs from MIMIC-IV-ECG with weights randomly initialized using truncated normal initialization (Figure 1). We used the Adam optimizer with an initial learning rate of 3×10–4, ℓ2-weight decay of 1×10–4, default coefficients of β1=0.9 and β2=0.999, and a cosine annealing learning rate scheduler. To reduce model overfit we used stochastic weighted averaging [Izmailov et al.] at each residual block and a dropout layer with P=0.5 for the fully connected layers. The model was trained using binary cross-entropy loss for multilabel classification with label smoothing of 0.05 for each output class. To improve generalizability we applied data augmentation by using random shifting, scaling, gaussian noise, CutOut [DeVries et al.], and lead dropout to the waveform. Fivefold cross-validation was used to assess robustness of the model during development. Models were trained for 100 epochs using an early stopping of 12 consecutive epochs with no improvement to the cross-validation macro AUC. The model with the highest validation AUC on the final cross-validation fold was selected for testing.

### *Appendix references*

Izmailov, P., Podoprikhin, D., Garipov, T., Vetrov, D., & Wilson, A. G. (2018). Averaging weights leads to wider optima and better generalization. arXiv preprint arXiv:1803.05407.

DeVries, T., & Taylor, G. W. (2017). Improved regularization of convolutional neural networks with cutout. arXiv preprint arXiv:1708.04552.
